# A Patient Presenting with Joint Deformities and ST-Elevation Myocardial Infarction

**DOI:** 10.3390/diagnostics15172254

**Published:** 2025-09-05

**Authors:** Emil J. Dąbrowski, Wiktoria U. Kozłowska, Patrycja O. Lipska, Marcin Kożuch, Sławomir Dobrzycki

**Affiliations:** Department of Invasive Cardiology, Medical University of Białystok, 15-089 Białystok, Poland

**Keywords:** coronary artery disease, gout, myocardial infarction

## Abstract

A 62-year-old man presented with ST-elevation myocardial infarction and advanced tophaceous gout, despite long-term urate-lowering therapy. His history included chronic kidney disease, hypertension, heart failure, and atrial fibrillation. Examination revealed severe joint deformities with multiple tophi. Coronary angiography showed multivessel disease with critical right coronary artery stenosis, treated with primary percutaneous coronary intervention. Following a Heart Team consultation, the patient was bridged with cangrelor and underwent urgent hybrid coronary artery bypass grafting and left atrial appendage occlusion. This case highlights the systemic burden of treatment-refractory gout, with progressive cardiovascular and renal complications. Tophaceous gout represents a distinct, high-risk phenotype associated with increased mortality and reduced quality of life. Despite standard therapies, this patient experienced continued disease progression, prompting referral for advanced treatment with pegloticase and canakinumab. Multidisciplinary care and personalized strategies are essential in managing severe, refractory gout with multi-organ involvement.

**Figure 1:**
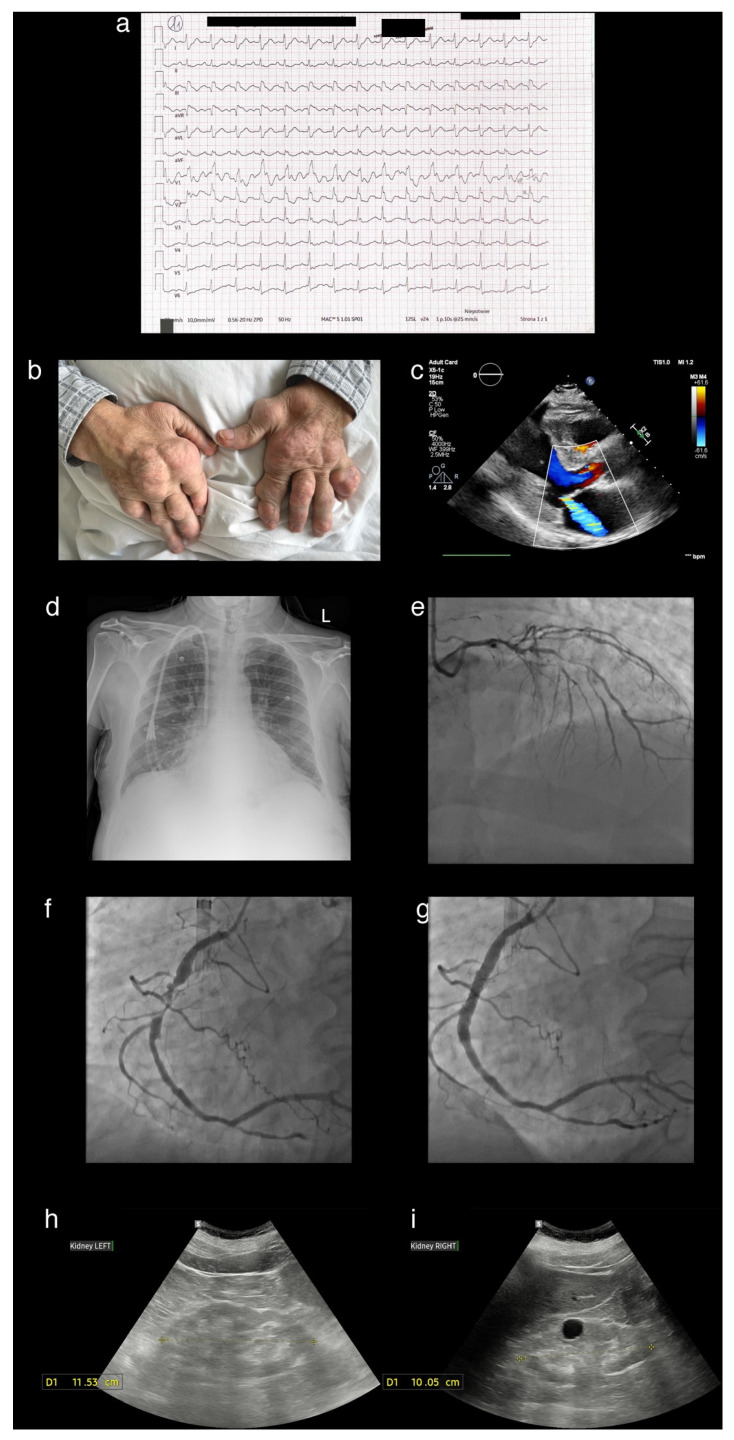
A 62-year-old man was admitted with inferior-wall ST-elevation myocardial infarction (panel (**a**)). His medical history included gout, diagnosed at age 32, chronic kidney disease, hypertension, heart failure, and paroxysmal atrial fibrillation. On examination, he exhibited multiple deformities of the small joints of the hands and feet, consistent with advanced tophaceous gout, which significantly impaired his quality of life, limiting both fine motor function and mobility. Surgical resections of chalk-like masses had been performed multiple times since 1996; however, new tophaceous nodules continued to develop (panel (**b**)—severe tophi affecting both hands with post-surgical scars after multiple tophectomy are visible). Despite long-term urate-lowering therapy with allopurinol, the patient reported recurrent gout flares, managed with colchicine and diclofenac. On admission, high-sensitivity troponin was 3025 ng/L, N-terminal pro-B-type natriuretic peptide > 35,000 pg/mL, serum urate was 5.4 mg/dL, and C-reactive protein was 104 mg/L. Electrocardiography demonstrated sinus rhythm at 84 bpm, intermediate axis, right bundle branch block, and pathological Q waves with ST-segment elevation in leads III and aVF (panel (**a**)). Urgent coronary angiography revealed severe multivessel coronary artery disease with critical right coronary artery stenosis, which was treated with primary percutaneous coronary intervention (panel (**c**)—advanced atherosclerotic lesions in the left coronary artery, including significant left main coronary artery stenosis; panels (**d**,**e**)—subtotally occluded right coronary artery, identified as infarct-related artery before and after primary percutaneous coronary intervention). Transthoracic echocardiography showed left ventricular hypertrophy with moderate ischaemic mitral regurgitation (panel (**f**)—long axis parasternal view of transthoracic echocardiography) and chest X-ray was notable for enlargement of cardiac silhouette, mild pulmonary congestion and a temporary hemodialysis catheter in the right subclavian vein (panel (**g**)). Over the past three years, he had been under regular nephrology follow-ups for progressive renal impairment and had recently commenced hemodialysis. Abdominal ultrasonography showed bilaterally atrophic kidneys with cortical thinning (panels (**h**,**i**)—ultrasonography revealing atrophic kidneys, nephrocalcinosis, and a simple cyst). Following a multidisciplinary Heart Team discussion, and in accordance with the contemporary European Society of Cardiology Guidelines [1], the patient was selected for hybrid revascularization and underwent urgent coronary artery bypass grafting with concomitant left atrial appendage occlusion. Coronary surgery was performed two weeks after the index myocardial infarction, following transfer from the Department of Invasive Cardiology to the Department of Cardiac Surgery. During the peri-operative period, he was bridged with cangrelor—short-acting P2Y12 inhibitor. After an uneventful recovery, he was discharged under the care of the Outpatient Cardiology Clinic and prescribed dual antiplatelet therapy (clopidogrel and aspirin). The patient was referred to the specialized center for consideration of pegloticase and canakinumab treatment qualification. Gout results from the deposition of monosodium urate crystals due to sustained hyperuricemia [2]. A cardinal manifestation of advanced disease is the formation of tophi, complex structures composed of monosodium urate crystals and associated inflammatory elements [3]. The tendency to form tophi suggests a distinct subgroup of patients with a more severe and progressive form of gout. Recent evidence supports the hypothesis that genetic polymorphisms may contribute to this predisposition [3]. Differential diagnosis should include calcium pyrophosphate deposition disease, a condition with distinct pathophysiology and limited to symptomatic treatment, as no disease-modifying therapies are currently available [4]. Beyond articular involvement, hyperuricemia promotes systemic inflammatory responses and is associated with cardiovascular diseases, chronic kidney disease, and increased mortality, even in the absence of conventional risk factors [2,5]. By increasing oxidative stress, impairing endothelial function, and amplifying vascular inflammation, elevated uric acid fosters atherosclerotic plaque development [6]. Consequently, hyperuricemia is linked with acute coronary syndromes, where it has been shown to predict both adverse in-hospital events and long-term cardiovascular outcomes [7]. Notably, gout increases the risk of mortality more than hyperuricemia alone, with the highest mortality rates observed in patients with tophaceous disease [8]. Effective urate-lowering therapy reduces the size and number of tophi, facilitating their dissolution and improving quality of life, which is frequently compromised in these patients. For individuals with tophaceous gout, current guidelines recommend a lower serum urate target (<5 mg/dL) to accelerate crystal dissolution and achieve complete resolution. In severe cases, crystal-proven tophaceous disease is unresponsive to conventional therapies, including combination regimens with pegloticase [9]. Pegloticase induces reduction in serum urate, accompanied by meaningful decreases in flare frequency, patient-reported pain, and resolution of tophi [3]. Despite long-term urate-lowering therapy, the patient developed severe complications of chronic gout, including marked joint deformity, and progressive renal and cardiovascular decline, which ultimately led to referral to a specialized center. These observations highlight the importance of personalized therapeutic strategies and vigilant monitoring for systemic organ involvement in patients with treatment-refractory disease.

## Data Availability

No new data were created or analyzed in this study.

## References

[1] Van Gelder I.C., Rienstra M., Bunting K.V., Casado-Arroyo R., Caso V., Crijns H.J.G.M., De Potter T.J.R., Dwight J., Guasti L., Hanke T. (2024). 2024 ESC Guidelines for the management of atrial fibrillation developed in collaboration with the European Association for Cardio-Thoracic Surgery (EACTS). Eur. Heart J..

[2] Du L., Zong Y., Li H., Wang Q., Xie L., Yang B., Pang Y., Zhang C., Zhong Z. (2024). Hyperuricemia and its related diseases: Mechanisms and advances in therapy. Signal Transduct. Target. Ther..

[3] Lawrence Edwards N., Singh J.A., Troum O., Yeo A.E., Lipsky P.E. (2019). Characterization of patients with chronic refractory gout who do and do not have clinically apparent tophi and their response to pegloticase. Rheumatology.

[4] Stack J., McCarthy G. (2021). Calcium pyrophosphate deposition (CPPD) disease—Treatment options. Best Pract. Res. Clin. Rheumatol..

[5] Kralisz P., Dąbrowski E.J., Dobrzycki S., Kozłowska W.U., Lipska P.O., Nowak K., Gugała K., Prokopczuk P., Mężyński G., Święczkowski M. (2025). Long-term impact of diabetes on mortality in patients undergoing unprotected left main PCI: A propensity score-matched analysis from the BIA-LM registry. Cardiovasc. Diabetol..

[6] Li K., Li K., Yao Q., Shui X., Zheng J., He Y., Lei W. (2023). The potential relationship of coronary artery disease and hyperuricemia: A cardiometabolic risk factor. Heliyon.

[7] Maloberti A., Biolcati M., Ruzzenenti G., Giani V., Leidi F., Monticelli M., Algeri M., Scarpellini S., Nava S., Soriano F. (2021). The Role of Uric Acid in Acute and Chronic Coronary Syndromes. J. Clin. Med..

[8] Disveld I.J.M., Zoakman S., Jansen T.L.T.A., Rongen G.A., Kienhorst L.B.E., Janssens H.J.E.M., Fransen J. (2019). Crystal-proven gout patients have an increased mortality due to cardiovascular diseases, cancer, and infectious diseases especially when having tophi and/or high serum uric acid levels: A prospective cohort study. Clin. Rheumatol..

[9] Richette P., Doherty M., Pascual E., Barskova V., Becce F., Castañeda-Sanabria J., Coyfish M., Guillo S., Jansen T.L., Janssens H. (2017). 2016 updated EULAR evidence-based recommendations for the management of gout. Ann. Rheum. Dis..

